# CO_2_ Efflux from Shrimp Ponds in Indonesia

**DOI:** 10.1371/journal.pone.0066329

**Published:** 2013-06-05

**Authors:** Frida Sidik, Catherine E. Lovelock

**Affiliations:** The School of Biological Sciences, The University of Queensland, St Lucia, Queensland, Australia; Dowling College, United States of America

## Abstract

The conversion of mangrove forest to aquaculture ponds has been increasing in recent decades. One of major concerns of this habitat loss is the release of stored ‘blue’ carbon from mangrove soils to the atmosphere. In this study, we assessed carbon dioxide (CO_2_) efflux from soil in intensive shrimp ponds in Bali, Indonesia. We measured CO_2_ efflux from the floors and walls of shrimp ponds. Rates of CO_2_ efflux within shrimp ponds were 4.37 kg CO_2_ m^−2^ y^−1^ from the walls and 1.60 kg CO_2_ m^−2^ y^−1^ from the floors. Combining our findings with published data of aquaculture land use in Indonesia, we estimated that shrimp ponds in this region result in CO_2_ emissions to the atmosphere between 5.76 and 13.95 Tg y^−1^. The results indicate that conversion of mangrove forests to aquaculture ponds contributes to greenhouse gas emissions that are comparable to peat forest conversion to other land uses in Indonesia. Higher magnitudes of CO_2_ emission may be released to atmosphere where ponds are constructed in newly cleared mangrove forests. This study indicates the need for incentives that can meet the target of aquaculture industry without expanding the converted mangrove areas, which will lead to increased CO_2_ released to atmosphere.

## Introduction

Soil is one of major sources of CO_2_ emissions to the atmosphere [1], [2], [3]. Soil respiration, determined by measuring the CO_2_ efflux from soil surface, is primarily from the respiration of soil organisms and roots [1], [3]. On a global scale, rates of soil respiration in vegetated biomes have a positive relationship with plant productivity, which contributes to soil metabolic activity [1]. In the absence of vegetation, e.g. when land is converted to aquaculture ponds, the microbial community plays a major role in soil respiration and can release large amounts of CO_2_ to the atmosphere [1], [4]. During pond construction and operation sediment carbon is increasingly exposed to air, microbial activity accelerates which may result in increases in CO_2_ efflux from the soil [5], [6].

Mangroves are known to be habitats that sequester and store significant amounts of carbon, referred as ‘blue’ carbon [7]–[10]. The carbon stored in mangroves is mostly found below ground, comprised of highly organic soils and roots [7], [11]. The removal of the mangrove forest (aboveground biomass) leads to reduction of carbon sequestration and the release of soil carbon stocks in the form of CO_2_ to the atmosphere [5], [6], [12]. Recent studies have provided global estimates of the CO_2_ efflux contribution to global greenhouse gas (GHG) emissions due to mangrove loss in order to assess the potential implications of continuing mangrove wetland conversion and the potential for GHG mitigation schemes [5], [6], [12], [13], yet there are few empirical studies of GHG emissions from aquaculture ponds that occur in converted mangrove areas.

The conversion of mangroves to aquaculture ponds has been a critical issue in Indonesia. With a cover of 3,112,989 ha of mangrove forests, which is the largest portion of remaining global mangrove cover [13], [14], Indonesia’s coasts also comprise extensive areas of aquaculture ponds [15], [16], [17]. Increasing shrimp production in the 1990s led to the expansion of mangrove forest conversion to aquaculture ponds at a rate of 3.67% per year [15]. Rapid conversion of mangrove forests to aquaculture ponds mainly occurred in regions in Sumatra and Kalimantan, however many of those areas have been abandoned in the past decades [15]. Recently, the government has begun to manage the abandoned areas and has expressed interest in increasing shrimp production through revitalisation of existing shrimp ponds [17]. There has also been a substantial effort to restore ponds to mangrove forests by both government and non-government organizations [15], [18]. But there is little information of the carbon emissions that are avoided through restoration, which could provide further incentives for restoration of non-productive ponds.

This study examined CO_2_ efflux from soil in an intensive shrimp farm in Bali, Indonesia. Shrimp aquaculture is one of major aquaculture activities in Bali. In this study we measured the CO_2_ efflux from the soil of the floors and walls of the shrimp ponds that were established 20 years ago. Furthermore, we estimated from these measurements a potential annual CO_2_ efflux from shrimp ponds using a dataset of aquaculture area in Indonesia. The results of this study have implications for initiatives aimed at preparedness for use of the clean development mechanism (CDM) and other emissions trading in countries with extensive aquaculture.

## Results

**Figure 1:**
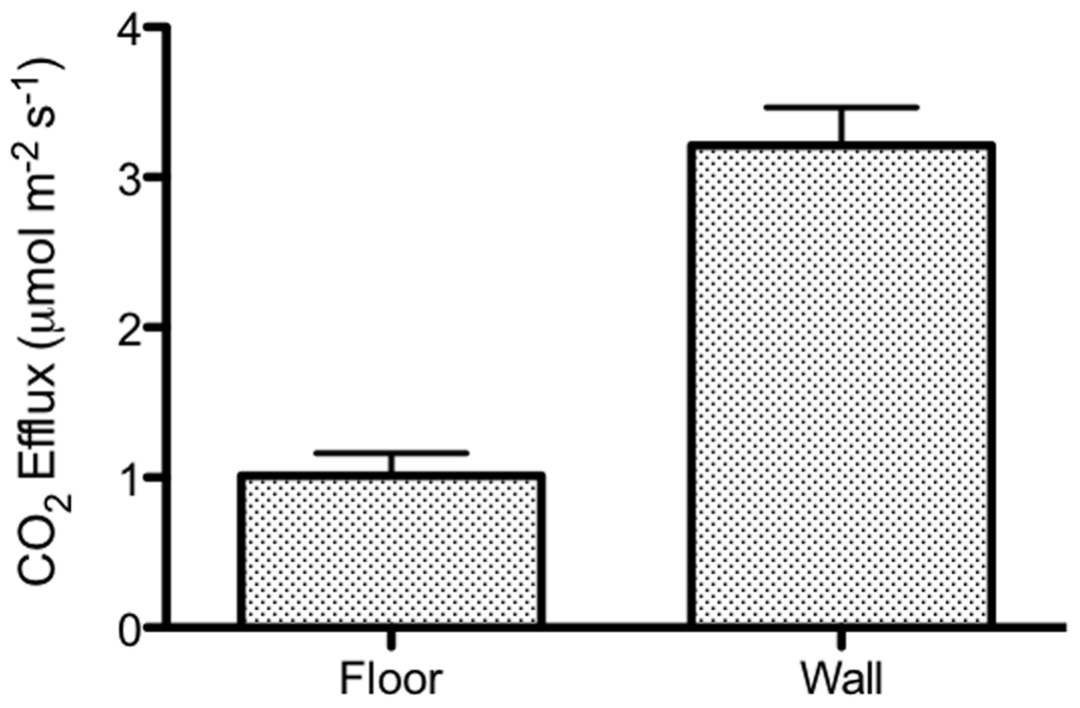
CO_2_ efflux from the floors and walls of shrimp ponds in Bali, Indonesia. The rates of CO_2_ efflux from the walls were significantly higher than pond floors (F_1,28_ = 25.66, P<0.0001).

Measures of CO_2_ efflux within shrimp ponds showed that rates of CO_2_ efflux from the walls were 3.15 µmol m^−2^ s^−1^ which exceeded emissions from the floors of the pond which were 1.15 µmol m^−2^ s^−1^ (Figure 1, F_1,28_ = 25.66, P<0.0001). Extrapolation of CO_2_ efflux rates to annual CO_2_ loss from shrimp ponds to atmosphere gave values of 4.37 kg CO_2_ m^−2^ y^−1^ (walls) and 1.60 kg CO_2_ m^−2^ y^−1^ (floors). Soil temperature varied significantly between floors and walls of the pond (F_1,28_ = 21.81, P<0.0001), ranging from 31.9°C to 37.0°C in the floors (mean of 34.5°C) and from 30.9°C to 45.1°C in the walls (mean of 40°C).

Using the above values we developed estimates of annual CO_2_ efflux from shrimp ponds in Indonesia. There are two published values within Indonesian government reports of the area of shrimp ponds in Indonesia [16], [17]. These gave a “low” estimate of CO_2_ emissions of 5.76 Tg y^−1^ and a “high” estimate of 13.95 Tg y^−1^.

## Discussion

Our measurements of CO_2_ efflux from the floors of shrimp ponds in Bali found lower rates of soil CO_2_ efflux than have been measured in mangroves that were cleared in Belize [5]. The lower CO_2_ efflux rates may be due to the lower carbon density in the Bali soils, which are mineral soils (0.019 g C cm^−3^), compared to the average mangrove soil carbon density in Indonesia and Southeast Asia and the highly organic peat soils in Belize [5], [19]. But we found higher rates of CO_2_ efflux from walls than pond floors, which increased the overall CO_2_ efflux per area from the ponds. High respiration in pond walls was coincident with warmer temperature in pond walls than pond floors. The structure of ponds, where soil was pushed up to form walls, allows the soil in the walls to receive high levels of solar radiation and high levels of aeration probably leading to increased rates of C oxidation [5]. Warmer temperatures stimulate microbial activity resulting in greater CO_2_ efflux from decomposition thereby increasing the release of CO_2_ to the atmosphere [1], [2], [5], [6], [20].

Our measured rates of CO_2_ efflux from shrimp ponds were within the range reported in previous work by Burford and Longmore (2001) who measured CO_2_ efflux from pond water surfaces rather than from the soil surface [4], [21]. CO_2_ efflux measured by Burford and Longmore (2001) [4] was highly variable which likely reflects variation in pond management, temporal factors or other biogeochemical factors [2], [4], [6], [13], [19].

Emissions profiles of land conversion may be variable, dependent on climatic and substrate factors, year and type of land uses [2], [4], [6], [13]. Studies of soil CO_2_ emissions from several forms of established land uses in converted Asian peat forest (Table 1) indicate comparable levels of CO_2_ emissions to shrimp aquaculture [22]–[25].

**Table 1:** Comparison of CO_2_ emissions from land uses linked to tropical forest loss.

Type of land conversion	Location	Carbon emission	Source
Shrimp ponds	Bali, Indonesia	1.60 kg CO_2_ m^−2^ y^−1^ (floors)	This study
		4.37 kg CO_2_ m^−2^ y^−1^ (walls)	
Mangrove clearing	Belize	2.9 – 10.6 kg CO_2_ m^–2^ y^–1^	Lovelock et al (2011)
Paddy field	Kalimantan, Indonesia	1.4 kg CO_2_ – C m^–2^ y^–1^	Hadi et al (2005)
Abandoned paddy field	South Kalimantan, Indonesia	∼1.2 – 1.5 kg CO_2_ – C m^−2^ y^−1^	Inubushi et al (2003)
Oil palm plantation	South Asia	∼0.75 – 1.1 kg CO_2_ m^−2^ y^−1^	Reijnders and Huijbregts (2008)
	Sarawak, Malaysia	1.5 kg CO_2_ – C m^−2^ y^−1^	Melling et al (2005)
Sago palm plantation	Sarawak, Malaysia	1.1 kg CO_2_ – C m^−2^ y^−1^	Melling et al (2005)
Rice-soybean rotation field	Kalimantan, Indonesia	2 kg CO_2_ – C m^−2^ y^−1^	Hadi et al (2005)

The values of CO_2_ efflux from pond floors were similar to other land uses, e.g. paddy fields, oil palm and sago palm plantation. CO_2_ emissions from recently constructed ponds in converted mangrove forests may be higher than presented here. Therefore the expansion of shrimp ponds and mangrove forest conversion could result in higher magnitudes of annual CO_2_ emissions from aquaculture compared to the findings from this study. In the economic analysis of the benefits of protecting mangroves for their carbon values, Siikamäki et al (2012) [19] assumed a loss of 27% (low end) and 90% (high end) of soil carbon once mangroves were converted and a mean soil carbon of 0.0418 g C cm^−3^ for Indonesia. Over 20 years loss of 27% carbon gives a CO_2_ emission rate of 2.2 kg CO_2_ m^−2^ y^−1^. Our data, from ponds in mineral soils, are consistent with this estimate.

The conversion of mangrove forest to aquaculture causes significant increases in CO_2_ efflux to the atmosphere, and thus strong incentives to preserve coastal wetlands are needed to avoid increasing CO_2_ released to atmosphere. Restoration and conservation of coastal wetlands are the primarily mechanisms proposed to reduce CO_2_ emission driven from mangrove loss [13]. However, application of these mechanisms is difficult in regions targeted for enhanced aquaculture production, as is the case in Indonesia [15], [17]. Revitalisation of existing shrimp ponds is an additional option to meet the goals of increased aquaculture productivity without expanding the converted area that leads to increasing CO_2_ released to atmosphere.

Our estimates of CO_2_ efflux from shrimp pond aquaculture may be improved by increasing the spatial and temporal sampling of CO_2_ emissions from aquaculture land use. Soil respiration in forested ecosystems varies with metabolic activity of tree roots, variation in temperature and water content of soils [2]. In aquaculture ponds live roots are eliminated and soils are saturated which may reduce variation in CO_2_ efflux. However the temperature of pond soils may vary widely which could be incorporated into more complex modelling of CO_2_ emissions from aquaculture ponds. Additionally, greater confidence in our scaling up requires improved estimates of the aerial extent of existing shrimp ponds in Indonesia [26]. This study used the area of existing aquaculture published in 2001 (438,010 ha) to calculate the “high” estimate of country-wide CO_2_ emissions from shrimp ponds [16]. We assumed that all aquaculture land was utilised for shrimp pond production as the data indicated that shrimp (*Paneous monodon*) culture dominated in aquaculture areas in Indonesia, second only to culture of milkfish (*Chanos chanos*). Our “high” estimate scenario however is likely an underestimate because conversion of mangrove forest to ponds was greater than were reported [15]. Additionally, our “low” estimate scenario, which used more recent data (2011) of existing working shrimp ponds in 22 districts in Indonesia of 180,844 ha [17], did not include previously converted ponds that are not currently in production. Improved documentation of areas of ponds and management of ponds would enhance our confidence of estimates of CO_2_ emissions contributed by aquaculture in Indonesia.

## Conclusion

The conversion of mangrove forests to shrimp ponds resulted in CO_2_ losses to the atmosphere for 4.37 kg CO_2_ m^−2^ y^−1^ from the walls and 1.60 kg CO_2_ m^−2^ y^−1^ from the floors of ponds. Our estimate of annual CO_2_ emission from shrimp ponds in Indonesia region was between 5.76 and 13.95 Tg y^−1^. These values are comparable to CO_2_ emissions from other land uses of converted lowland forests. The CO_2_ emission released to atmosphere might be higher than we report here if ponds are constructed in newly cleared mangrove forests. Knowledge of the amounts of CO_2_ released from shrimp ponds may contribute to preparedness for use of the clean development mechanism (CDM) and other emissions trading schemes in countries, particularly Indonesia, which have made a commitment to protect mangrove forests concurrently with commitments to meet targets of high production in the aquaculture industry.

## Materials and Methods

This study was conducted in Perancak estuary, Bali, Indonesia (8° 23' 40" S, 114° 37' 39" E). The area is a coastal plain associated with the Perancak River that is comprised of a mix of paddy fields, mangrove forests and aquaculture ponds. The estuary is characterised by sedimentary limestones and alluvial platforms. The soils are related to the volcanic stratigraphy derived from the Batur volcano [27], [28]. Soil organic carbon contents in this area, measured by Sidik et al (unpublished), are 0.019 g C cm^−3^ in the mangrove forests. In the 1990s, huge areas of mangrove forests were converted to intensive shrimp ponds, however, there is no literature that provides accurate information of the extent of mangroves cleared for shrimp ponds in the area. Since early 2000, these aquaculture activities have been diminished due to the global economic crisis and diseases, which have resulted in numerous shrimp ponds ceasing to be in production.

The measurements of CO_2_ efflux were made on the 24 and 25 November 2012. We made measurements between 10.30 am and 2 pm local time sampling the floors and walls of each pond twice during the measurement campaign. We selected three working shrimp ponds, which were about 20 years old. All the shrimp ponds were located in the same farm with similar soils and pond management. The area of each pond was about 2000 m^2^ with a depth of 1.5 m. A day before the measurements of CO_2_ efflux the ponds were drained. Ponds in this farm are usually stocked with *Panaeus vanamei* and aerated with paddlewheels. We measured the CO_2_ efflux from the soil in floors and walls of these ponds. CO_2_ efflux from soils was measured using a LiCor 6400 portable photosynthesis system configured with the LiCor soil CO_2_ flux chamber (LiCor Corp, Lincoln, NE, USA) inserted 0.5 cm into the soil. Soil temperature was measured at 2 cm depth simultaneously with CO_2_ efflux. We conducted five measurements in the floor and walls of each pond. The locations of the measurements within each pond were randomly distributed over the floors and walls and were at least 1 m apart from each other. The surface of the floor of the pond was scraped to remove the microalgal film prior to each measurement after which the chamber was placed on the surface of the sediment. Differences in CO_2_ efflux rates between floors and walls were assessed using ANOVA.

We extrapolated to CO_2_ efflux for shrimp ponds in Indonesia by multiplying our measurements scaled up to an annual rate by the reported area of shrimp ponds. The areas of shrimp ponds were derived from recent information published by Indonesian government [16], [17]. As uncertainties existed in the available data, we used a conservative approach to estimate the CO_2_ efflux from shrimp ponds [26]. We multiplied the mean of CO_2_ efflux from ponds by two different estimates of pond areas to generate “high” and “low” estimates of CO_2_ emission from ponds. The lower area came from published information from the Ministry of Marine Affairs and Fisheries [17] and the high estimate from literature [16] derived from the Ministry of Agriculture. Our surveys of the area indicated that pond floors occupied approximately 90% of the shrimp pond footprint and walls about 10%. However, walls were three dimensional, typically 1.5 m high and 1 m wide at the top (4 linear meters). We therefore estimated total CO_2_ efflux from floors and walls as (0.9 x floor efflux) + (0.1 x wall efflux x 4) to incorporate the three dimensional nature of the walls.

### Ethics Statement

The field measurement was undertaken in a private shrimp farm in Perancak, Bali, with the permission from the owner.
